# Are There Differences in the Anthropometric, Hemodynamic, Hematologic, and Biochemical Profiles between Late- and Early-Onset Preeclampsia?

**DOI:** 10.1155/2018/9628726

**Published:** 2018-03-01

**Authors:** Márcia Aires Rodrigues de Freitas, Alice Vieira da Costa, Luciana Alves de Medeiros, Mario da Silva Garrote Filho, Angélica Lemos Debs Diniz, Nilson Penha-Silva

**Affiliations:** ^1^Faculty of Medicine, Federal University of Uberlândia, Uberlândia, MG, Brazil; ^2^Institute of Genetics and Biochemistry, Federal University of Uberlândia, Uberlândia, MG, Brazil

## Abstract

Preeclampsia (PE) is classified as early-onset PE (EOPE) and late-onset PE (LOPE) when present before or after 34 weeks of gestation, respectively. This transversal study aimed to investigate the differences and possible associations existing in the anthropometric, hemodynamic, hematologic, and biochemical profiles of late- and early-onset preeclampsia. The study included 65 volunteers admitted to a tertiary hospital in Brazil: 29 normotensive and 36 with preeclampsia (13 with EOPE and 23 with LOPE). Pregnant women with LOPE presented greater weight gain and borderline increase in body mass index at the end of gestation in relation to the other groups, which is compatible with the metabolic origin, associated with obesity, attributed to this form of the disease. Pregnant women with EOPE presented a borderline reduction in the number of erythrocytes and a significant decrease in the number of platelets, in addition to a significant increase in reticulocytes, serum iron, and ferritin when compared to normotensive pregnant women and pregnant women with LOPE. A significant increase in osmotic stability of erythrocytes was observed in the EOPE group in relation to other groups. Hemodynamic analysis by Doppler ultrasonography of the ophthalmic artery showed that both groups of pregnant women with PE presented alterations compatible with the occurrence of hyperflow in the orbital territory. These hemodynamic changes were associated with changes in hematimetric indices.

## 1. Introduction

Preeclampsia (PE) is a complex multifactorial syndrome with etiology not yet established, which affects between 5 and 7% of pregnancies worldwide [1, 2]. The diversity of repercussions in the pregnant woman and in the fetus has been investigated in light of its classification in early-onset PE (EOPE) and late-onset PE (LOPE), when present before and after 34 weeks of gestation, respectively [3]. This classification seems to reflect etiopathogenic mechanisms that begin at different moments of gestation. Some authors highlight the occurrence of differences between these two types of PE in terms of the anthropometric, hematologic, and biochemical profiles of the pregnant woman and the maternal-fetal outcomes so that how earlier is its onset, worse is the maternal-fetal outcome [3, 4]. This would justify, at least in part, the theory that EOPE and LOPE would have different pathophysiologies; however, there are controversies on the subject [5].

In PE, neurologic changes are the most serious and potentially fatal maternal complications. Epidemiological studies have shown that neurologic complications occur more frequently in EOPE than in LOPE [6, 7]. Notably, this suggests that EOPE is the most severe form of PE, promoting neurologic complications due to central vascular alterations [8–11]. However, the factors that contribute to neurologic involvement during EOPE are not well known [7].

The maternal cerebral vasculature can be studied by Doppler of the ophthalmic artery (OA). Doppler velocimetric findings of OA can be extrapolated to the cerebrovascular circulation [12–14] because of origin in the internal carotid, which is responsible by much of the cerebral circulation and by their embryological and functional similarities with small-caliber cerebral vessels' responsibility [15]. Previous studies on the subject have identified in PE the presence of cerebrovascular hyperflow, which many authors have termed maternal centralization, in analogy to what occurs in the fetal cerebral circulation in situations of hypoxia [12, 16, 17]. Several authors have previously described the hemodynamic profile of OA in pregnant women with PE [17–19], but there are no specific publications on this vascular pattern in EOPE and LOPE.

The oxidative aggression to the endothelium, leading to inflammation and culminating in an increase in vascular resistance, with consequent elevation in blood pressure, is one of the mechanisms accepted to explain the installation of PE [20, 21]. Blood flow control is directly associated with tissue perfusion, and the erythrocyte has an active function in this process. For the good performance of its functions, the erythrocyte must be able to undergo extensive passive deformation, without suffering lysis, when passing through small-capillary capillaries. This is possible, thanks to the viscoelasticity of the erythrocyte membrane.

It is known that the relative content of cholesterol and phospholipids (with polyunsaturated fatty acids), which depends on blood lipid levels, is essential for maintaining the fluidity, deformability, stability, and functionality of erythrocytes [22]. Since PE is related to dyslipidemia, oxidative stress, and hematologic and vascular changes [23–25], it is possible that these changes are associated with each other. Within this context, it makes perfect sense to consider the participation of the erythrocyte in the etiology of PE/eclampsia, even because it has been associated with disease progression, particularly due to the strong association between this cell and oxidative stress [26, 27].

In this study, the possible association of alterations in erythrocyte behavior with PE will be investigated through the analysis of osmotic stability. The analysis of the erythrocyte behavior in a hyposmotic gradient is a powerful method that reflects the deformability [28], stability [29, 30], and the membrane composition of these cells, allowing the investigation of the influences of the lipid blood level [31] oxidative aggression [26], among other factors. Although there are already studies on the osmotic stability of erythrocytes in both pregnancy and PE, they are usually presented as a percentage of hemolysis as a function of time [32] and lysis ratio at specific salt concentration [26]. In this work, the osmotic stability of erythrocytes was evaluated by statistical treatment of the hyposmotic gradient lysis curve, using variables and indices that have been very useful in the evaluation of several other physiologic or pathologic conditions [29, 33].

Faced with the need to better understand the differences between EOPE and LOPE, this study compared anthropometric, hemodynamic, biochemical, hematologic, and osmotic stability profiles of red blood cells between these two types of PE.

## 2. Materials and Methods

### 2.1. Population

The study project was registered and approved by the Research Ethics Committee of the Federal University of Uberlândia (CEP-UFU) under the number CAAE 23236614.4.000.5152. All patients signed a Free and Informed Consent Form. The study included 65 pregnant women admitted to the Clinical Hospital of the Federal University of Uberlândia between December 2014 and June 2017. Pregnant patients without pathologic changes in pressure and obstetric routine exams, without signs of labor, and gestational age greater than or equal to 39 weeks were selected to form the control group (*n* = 29). The problem group consisted of pregnant patients who met the criteria defined for diagnosis of PE (*n* = 36) by the American College of Obstetricians and Gynecologists [34].

The problem group was stratified into subgroups of pregnant women with EOPE (*n* = 13) or LOPE (*n* = 23), according to the gestational age of onset of signs/symptoms of the disease [35].

All the pregnant women included in the study took folic acid supplementation (5 mg/day) in the first trimester and started supplementation of elemental iron (40 mg/day) at the second trimester of pregnancy.

Volunteers with a history of thyroid diseases, erythrocytopathies, chronic hypertension, autoimmune diseases, diabetes, and renal diseases, and volunteers with current use of anticonvulsants, antidepressants, magnesium sulfate, tobacco, alcohol, and/or other drugs, and patients with gestations associated with congenital (toxoplasma and cytomegalovirus) and sexually transmitted diseases, twin pregnancy, fetal malformation, restriction of fetal growth not associated with hypertensive disease, and natimortality were excluded from the study.

### 2.2. Blood Collection

Blood samples were collected by venipuncture in tubes with K_3_EDTA or without anticoagulant (Vacutainer, Becton Dickinson, Juiz de Fora, Brazil) and were stored at 0–4°C until the time of each test. Blood collection was performed on admission of the volunteers at the Clinical Hospital of the Federal University of Uberlândia (CH-FUU).

### 2.3. Determination of Osmotic Stability of Erythrocytes

A duplicate set of test tubes (24) containing 1 mL of 0.1–1.0 g/dL NaCl solution (Labsynth, Diadema, Brazil) was preincubated at 37°C in a thermostated water bath (Marconi, Model MA 184, Piracicaba, SP, Brazil) for 10 minutes. After addition of 20 *μ*L of whole blood, the tubes were gently shaken, reincubated for 30 minutes and then centrifuged at 1600 ×g in a centrifuge (model CFR15XRII, Hitachi Koki, Hitachinaka, Japan) for 10 minutes, for removal of the supernatant and analysis of the amount of hemoglobin released in the hemolysis by the absorbance reading at 540 nm in a UV-Vis spectrophotometer (UV1650TC model, Shimadzu, Japan).

The graphs of A540 as a function of the NaCl (*X*) concentration were adjusted by nonlinear sigmoidal regression, according to the Boltzmann equation:

(1) $$\begin{array}{c} A_{540}=\frac{A_{\max}-A_{\min}}{1+e^{\left(X-H_{50}\right)/dX}}+A_{\min}, \end{array}$$

where *A*_max_ and *A*_min_ represent, respectively, the maximum and minimum plateaus of *A*_540_, *H*_50_ is the concentration of NaCl capable of promoting 50% hemolysis, and *dX* represents 1/4 of the change in NaCl concentration responsible for 100% hemolysis [30].

The analysis of osmotic stability of erythrocytes was performed up to 24 hours after blood collection.

### 2.4. Doppler Ultrasonography of the Ophthalmic Artery

The OA Doppler was performed on ultrasound equipment (model SonoAce 8800MT, Medison, Japan), by a single examiner with more than 10 years of experience. OA was insonated in the medial region of the optic nerve with a linear transducer at 7–10 MHz frequency, 50 Hz filter, 5 kHz pulse repetition frequency, and 7 mm sample volume [12, 13]. Only one eye was examined because previous studies did not show significant differences between the eyes [12, 36].

The study of Doppler velocimetry of the ophthalmic artery was performed on admission of volunteers in CH-FUU.

### 2.5. Determination of Hematologic and Biochemical Parameters at the Third Trimester of Gestation

Hematologic parameters were obtained using an automated system (Sysmex K4500, Sysmex Corporation, Mundelein, IL, USA). These parameters with their respective reference values (third trimester of gestation) included erythrocyte count (RBC), 2.71–4.43 × 10^6^/mm^3^; hematocrit (Ht), 28.0–40.0%; hemoglobin (Hb), 9.5–15.0 g/dL; mean corpuscular volume (MCV), 81–99 fL; mean corpuscular hemoglobin (MCH), 29–32 pg/cell; mean corpuscular hemoglobin concentration (MCHC), 31–36 g%; red-cell distribution width (RDW), 12.7–15.3%; platelet count (Plt), 146–429 × 10^3^/mm^3^; and mean platelet volume (MPV), 8.2–10.4 fL [37, 38].

Biochemical parameters were measured by an automated analyzer (Architect c8000, IL, USA) and included triglycerides (TGC), 131–453 mg/dL; total cholesterol (t-C), 219–349 mg/dL; high-density lipoprotein cholesterol (HDL-C), 48–87 mg/dL; low-density lipoprotein cholesterol (LDL-C), 101–224 mg/dL; very-low-density lipoprotein cholesterol (VLDL-C), 21–36 mg/dL; lactate dehydrogenase (LDH), 82–524 U/L; alanine aminotransferase (ALT), 2–25 U/L; aspartate aminotransferase (AST), 4–32 U/L; urea (U), 3–11 mg/dL; creatinine (Cn), 0.4–0.9 mg/dL; uric acid (UA), 3.1–6.3 mg/dL; human serum albumin (HSA), 2.3–4.2 g/dL; sodium (Na^+^), 130–148 mEq/L; potassium (K^+^), 3.3–5.1 mEq/L; indirect (i-B), 0.1–0.5 mg/dL, and total bilirubin (t-B), 0.1–1.1 mg/dL; serum iron (Fe), 30–193 *µ*g/dL; and ferritin, 0–116 ng/mL [39, 40].

The evaluation of the presence of proteinuria and its quantification were performed on urine reagent tape (Labtest, Lagoa Santa, MG, Brazil).

### 2.6. Statistical Analysis

The normality of the data set was evaluated by the Shapiro–Wilk test. Analysis of variance (ANOVA) test followed by Bonferroni post hoc test and Kruskal–Wallis test followed by Dunn–Bonferroni post hoc test were used to compare the groups of normal and nonnormal variables, respectively. Differences associated with *p* values < 0.05 and 0.05 < *p* < 0.10 were considered statistically significant and borderline, respectively. Most variables were not normally distributed, and therefore the Spearman correlation test was used to find associations between studied variables. All analyses were performed using Origin 8.5 professional (MicroCal, Northampton, MA, USA) and SPSS 15.0 (SPSS Inc., Chicago, IL, USA) software packages.

## 3. Results

**Figure 1:**
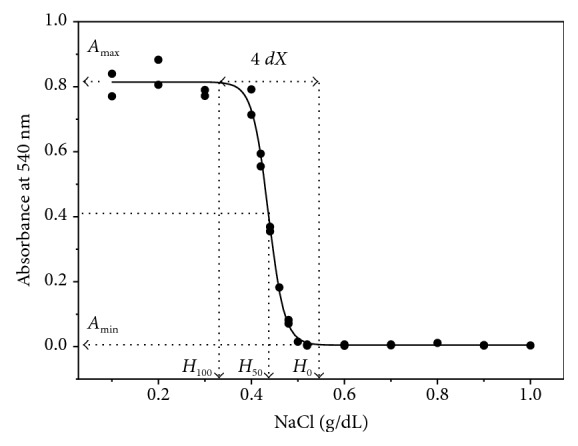
Typical curve of osmotic stability of erythrocytes. The lower plateau defines the *A*_min_ variable, which represents the amount of basal hemolysis present in the blood sample of each study volunteer. The decrease in the tonicity of the medium is associated with increased lysis of erythrocytes and release of hemoglobin in the solution, resulting in increased absorbance at 540 nm with formation of a curve of sigmoidal nature whose upper plateau defines the *A*_max_ variable, which represents the occurrence of 100% hemolysis. The curve passes through an intermediate point defining the *H*_50_ variable, which represents the concentration of NaCl required to promote 50% hemolysis. The saline concentration at the starting point of the curve defines the *H*_0_ variable, which is the saline concentration required to initiate *in vitro* hemolysis and which can be calculated by the formula *H*_0_ = *H*_50_ + 4 *dX*/2. The saline concentration at the point where the *in vitro* lysis reaches its maximum plateau defines the *H*_100_ variable, which represents the saline concentration necessary to promote the total lysis of the red blood cells, being calculated by the formula *H*_100_ = *H*_50_ − 4 *dX*/2. The *dX* variable represents a quarter of the variation in saline concentration required to promote 100% hemolysis.

Figure 1 shows a typical sigmoid curve obtained in the determination of erythrocyte membrane stability variables. The parameters *H*_0_, *H*_50_, and *H*_100_ are proportional to the osmotic fragility of the erythrocytes. Therefore, their inverse forms, 1/*H*_0_, 1/*H*_50_, and 1/*H*_100_, are those which effectively represent the osmotic stability of those cells. *dX* is effectively a variable of osmotic stability of erythrocytes, and therefore the *dX*/*H*_50_ and *dX*/*A*_min_ ratios are directly proportional to the erythrocyte membrane stability since *A*_min_ and *H*_50_ have inverse relationships with the stability of these cells.

**Figure 2:**
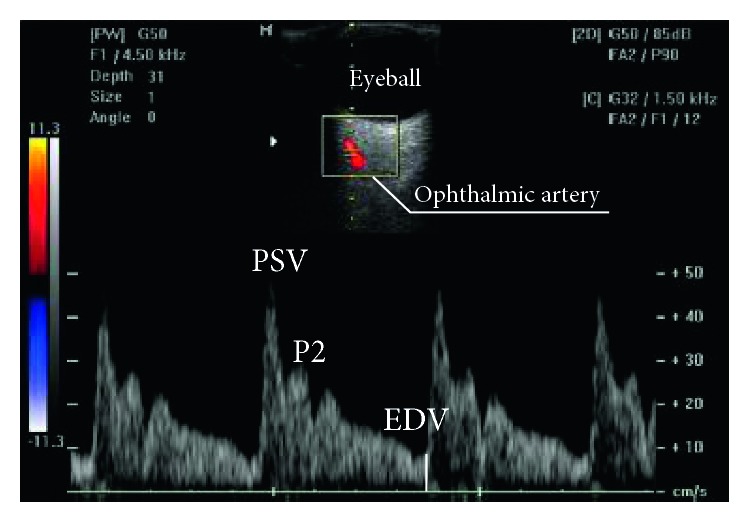
Doppler velocimetry of ophthalmic artery (OA), with identification of the pulse wave velocity (PWV) of the OA. The *X*-axis represents the time (seconds), and the *Y*-axis represents the flow velocity (cm/s). A rapid increase in velocity is observed, with a peak systolic velocity (PSV), followed by a rapid fall, and a new velocity rise with the formation of a second rounded systolic peak (P2), followed by the aortic notch, which closes the systolic cycle. Then, there is an increase in velocity with formation of the diastolic phase of the PWV, which ends with the end-diastolic velocity (EDV). The P2/PSV ratio is referred to as peak ratio [17].

**Figure 3:**
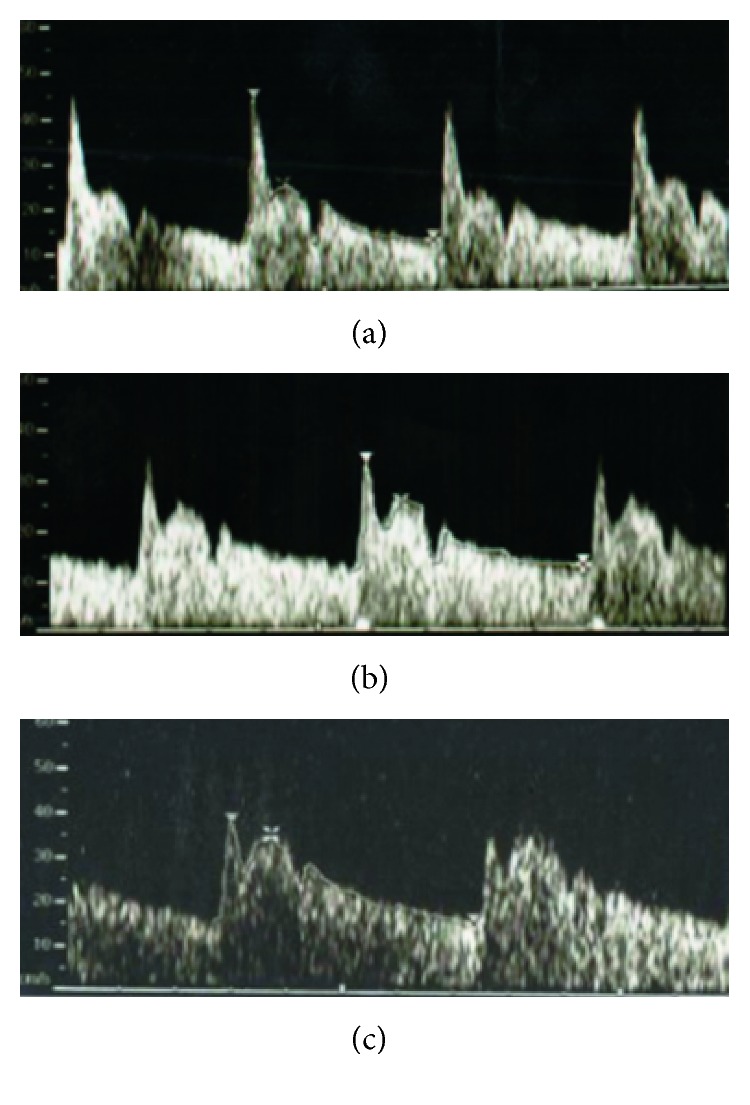
Representation of Doppler velocimetry of ophthalmic artery in a normotensive volunteer (PR = 0.56; P2 = 24.25) (a) and in volunteers with late-onset preeclampsia (LOPE: PR = 0.78; P2 = 25.24) (b) and early-onset preeclampsia (EOPE: PR = 0.93; P2 = 35.23) (c). PR is the P2/PSV ratio, and P2 is the second peak systolic velocity.

Figure 2 is a schematic representation of the variation of blood flow velocity in OA as a function of the duration time of a cardiac cycle. At each ejection of blood from the left ventricle (systole), a pulse pressure, which is represented by a rapid upward curve, occurs, generating the peak systolic velocity (PSV), which is followed by sudden drop, and a secondary elevation of velocity, generating a second systolic peak (P2) and a notch (aortic notch) before the end of the systolic cycle. As the vascular diameter returns to normal, the accumulated energy provides a potential needed to promote continuous flow during diastole, which is represented in the graph by rise of the speed, followed by slow deceleration and finishing at the peak of end-diastolic velocity (EDV). The interpretation of the graphical representation of an OA Doppler occurs by the analysis of the pulse wave velocity (PWV), the velocity peaks, the average velocity of a cardiac cycle (*V*_mean_), and the indexes that relate the systolic and diastolic velocities: pulsatility index (PI), given by (PSV-EDV)/*V*_mean_; resistance index (RI), given by (PSV-EDV)/PSV; and peak ratio (PR), given by P2/PSV.  Figure 3 shows typical Doppler velocimetry results observed in OA of normal pregnant women (3(a)) and pregnant women with preeclampsia in the present study (3(b) and 3(c)). In PE, a specific alteration in the morphology of the wave, which acquires the shape of hump, is observed due to the increase in amplitude and rounding of P2, indicating the presence of hyperflow in orbital territory. The elevation of PR and P2 indicates hyperflow in orbital territory. On the other hand, the decrease in IP and IR represents an increase in the flow velocity at that location [12].

Table 1 shows the values obtained for the main variables analyzed in the three groups of this study, presented as frequency (categorical variables), median ± IQR (variables without normal distribution), and mean ± SD (normal distribution).

In relation to the anthropometric variables, there was a significant increase in weight gain and a borderline increase in BMI during gestation in the group of pregnant women with LOPE in relation to the control (C) group. The weight gain during pregnancy was also significantly higher in the LOPE group than in the EOPE group. In fact, the deliveries of the EOPE group volunteers were very premature, according to World Health Organization criteria, with fetal weight 48% and 37% lower than in C and LOPE groups, respectively. The weight of the placenta in the EOPE group was also significantly lower than in the other groups.

Regarding blood cell counts, there was a borderline decrease in the erythrocyte count and a significant increase in the reticulocyte index in the EOPE group when compared to the C group. A significant reduction in platelets was also observed in the EOPE group in relation to the C group.

As for the liver function biomarkers, blood levels of ALT, AST, and LDH were higher in the LOPE and EOPE groups than in the C group. Blood levels of LDH were also significantly higher in the EOPE group than in the LOPE group.

The changes observed in these biomarkers make sense in relation to the lower levels of HSA observed in the volunteers of the EOPE group both in relation to the C group and the LOPE group.

In the category of nitrogen excretion products, urea and uric acid levels were significantly higher in the EOPE and LOPE groups than in the C group and were also higher in EOPE group than in LOPE group. The creatinine levels were higher only in the EOPE group than in the C group. There were no significant differences in the levels of indirect and total bilirubin between the groups.

Regarding electrolytes, plasma levels of Na^+^ were significantly lower in the EOPE group than in the LOPE and C groups, while K^+^ levels were significantly higher in EOPE group than in the C group.

Serum iron and ferritin levels were significantly higher in the EOPE and LOPE groups than in the C group and also higher in EOPE group than in the LOPE group.

As for Doppler velocimetric variables, there were significantly lower values of resistance index (RI) and pulsatility index (PI) of the OA Doppler in the EOPE and LOPE groups when compared to the C group. The volunteers of the EOPE group also presented significantly higher values of P2 than C group. The PR values of EOPE and LOPE groups were significantly higher in relation to the C group.

Regarding the membrane stability variables, the values of 1/*H*_50_ of the EOPE group presented a significant increase in relation to the C group and a borderline elevation in relation to the LOPE group. The EOPE group presented a borderline reduction of *H*_0_ and a significant reduction of *H*_100_ in relation to C group, as well as a borderline reduction of *H*_100_ in relation to the LOPE group.

In order to determine if the aforementioned differences would have been driven by the occurrence of growth restriction, the statistical analyses applied to the data in Table 1 were redone after the withdrawal of the volunteers with newborns whose growth was below the 10th percentile (results not shown), with probable etiology associated with preeclampsia. In total, 3 cases were withdrawn in the EOPE group and 1 in the LOPE group. Significant statistical differences were preserved in this new analysis, except for reticulocyte index and parameter *H*_100_. Differences with borderline significance were also preserved, except for the number of erythrocytes and the membrane stability variable *H*_0_.

The matrices for the correlations between all pairs of variables in the groups of pregnant women (normotensive, LOPE, and EOPE, resp.) are not presented in this study, but the Spearman's correlation coefficient (*ρ*) values, along with their respective *p* values, were mentioned in the discussion, whenever they were useful to support findings reported here.

## 4. Discussion

In the present study, the EOPE group presented the worst obstetric results when compared to the other groups, with very premature births, lower fetal and placental weights, and greater maternal systemic impairment, demonstrated by significant changes in hematological, biochemical, and Doppler velocimetric indices of AO. On the other hand, the LOPE group had a full-term birth, fetal weight close to 3 kg and placental weight without significant difference in relation to C group, and lower maternal systemic compromise when compared to the EOPE group.

Differences in gestational outcomes and maternal involvement suggest the presence of two distinct clinical phenotypes for PE [1, 41]. According to some authors, LOPE, described as its mild form, is associated with normal or slightly altered placentation, with clinical manifestations resulting from a previous endothelial compromise, which is exacerbated by pregnancy, and therefore, this form of the disease is called maternal PE. On the other hand, EOPE, a determinant of most serious cases, reflects an inadequate placentation, with the release of antiangiogenic and prooxidant factors, responsible for the promotion of endothelial lesion, and for this reason, this form of the disease is referred to as placental PE [42]. Endothelial dysfunction is the common pathway to all forms of presentation of the disease [5]. In PE, there is an imbalance between pro- and antioxidant factors, which characterizes the state of oxidative stress and can be proven by the elevation of biomarkers of lipid peroxidation in relation to normotensive pregnant women [26, 43].

Erythrocytes are quite vulnerable to reactive oxygen species (ROS) since their polyunsaturated fatty acids are susceptible to lipid peroxidation [44, 45]. This process leads to changes in the lipid bilayer of the erythrocyte membrane, affecting its fluidity and stability. With lysis, the iron released from hemoglobin favors the formation of free radicals superoxide (O2^•^) and hydroxyl (HO^•^), thus amplifying the cascade of oxidative damages. This is why membrane stability of erythrocytes should be a determining factor in the progression of preeclampsia [26].

Previous studies indicate that both gestation and PE are associated with changes in blood rheological behavior [46–51]. Although PE was previously associated with increased osmotic fragility of erythrocytes [43], in this study the EOPE group showed a significant increase in membrane osmotic stability in relation to the C group and a borderline increase in relation to the LOPE group. These results suggest that EOPE and LOPE actually have different etiologies. As osmotic stability is a good indicator of the extent of cellular impairment by lipid peroxidation [26], it is possible that this change in erythrocyte behavior is due to exacerbation of oxidative stress. The *in vivo* exacerbation of this oxidative aggression to erythrocytes would reduce their ability to deform and decrease their longevity, increasing their removal rate, the bilirubin production and the ferritin circulating levels, with consequent decrease in erythrocyte counts and acceleration of erythropoiesis, which is trivially associated with increased reticulocyte count. In fact, the EOPE group showed lower counts of red blood cells, higher levels of ferritin, and increased reticulocyte index.

In this complex situation, the involvement of erythrocytes in the pathophysiology of PE also has other implications that can be inferred from correlations presented by blood variables. Although the deformability of the erythrocyte membrane is related to its fluidity, which is impaired by oxidative stress, it is also influenced by cytoplasmic viscosity, which in turn is regulated by mean corpuscular hemoglobin concentration (MCHC) and intracellular volume of water. The cytoplasmic viscosity of the erythrocyte rises with increasing hemoglobin concentration such that excessive elevation will disturb the deformability of the erythrocyte. Thus, MCHC reference values reflect physiologic patterns of viscoelasticity of erythrocytes. In this sense, the negative correlation observed between MCHC and the stability variable *dX*/*A*_min_ in the control group (*ρ* = −0.392; *p*=0.047) should be meaning that, within the physiologic reference range of this variable, lower MCHC values are associated with greater osmotic stability, certainly because lower hemoglobin levels mean less impact of osmotic pressure in the promotion of *in vitro* lysis of the cells of normotensive pregnant women. For some reason not yet understood, in the LOPE group, the positive correlation of MCHC with the erythrocyte membrane stability was manifested in relation to the *H*_50_ (*ρ* =  +0.465; *p*=0.039) and *H*_100_ (*ρ* = +0.647; *p*=0.002) variables. But the positive nature of this association should mean that, also in LOPE, lower hemoglobin concentrations are associated with greater osmotic stability of erythrocytes. Indeed, the MCHC values should remain in a critical range to avoid compromising the stability and deformability of red blood cells.

**Figure 4:**
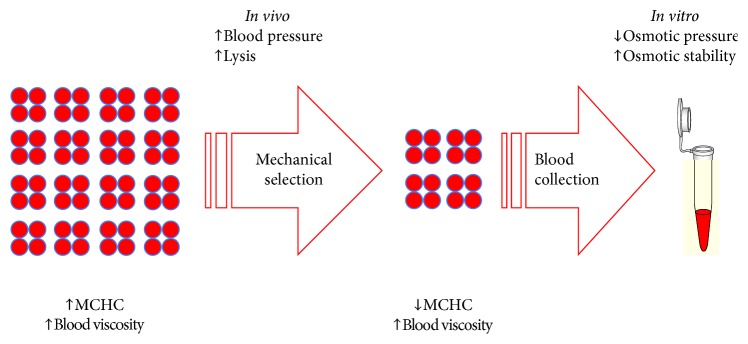
Mechanical selection and increased osmotic stability of erythrocytes in preeclampsia.

Although the osmotic stability of erythrocytes *in vitro* does not strictly reflect erythrocyte stability *in vivo*, since the hyposmolar conditions that lead to the *in vitro* hemolysis will not occur *in vivo*, it helps to understand erythrocyte behavior *in vivo*, where a broader set of mechanisms is at work. *In vivo*, the mechanical friction of erythrocytes with vessel wall, especially in smaller caliber capillaries, is a more relevant threat to the maintenance of erythrocyte stability. This mechanical aggression is aggravated by an increase in blood pressure. The influence of pressure on blood rheology is associated with an increase in the rate of removal and elevation of erythrocyte generation. The strong positive correlations observed between *dX* with SBP (*ρ* = +0.865; *p*=0.006) and DBP (*ρ* = +0.740; *p*=0.036) and *dX*/*H*_50_ with SBP (*ρ* = +0.636; *p*=0.036) and DBP (*ρ* = +0.664; *p*=0.026) reveal that the increase in blood pressure is also associated with the increase in erythrocyte stability *in vitro*, certainly due to an increase in the proportion of reticulocytes due to increase in the mechanical aggression by arterial hypertension on erythrocytes weakened due to exacerbation of oxidative aggression on their membrane lipids. The high pressure must have contributed to the removal of the most fragile cells such that the *in vitro* analysis is using only the more stable cells, which are remnants of a process of mechanical selection of stability. The negative and strong correlations of MCHC with DBP (*ρ* = −0.851; *p* < 0.001) and SBP (*ρ* = −0.911; *p* < 0.001) in the EOPE group indicate a very strong association between increased blood pressure and decline in MCHC. This should mean that this decline in MCHC is associated with change in the dynamics of the formation and removal of red blood cells. High MCHC values are associated with increased viscosity, which, in view of the elevation in blood pressure, would compromise the deformability and stability of the erythrocyte and consequently the dynamics of gas exchange in the pregnant woman. As smaller MCHC values were related to osmotically more stable erythrocytes in pregnant women with LOPE, as stated earlier, this suggests that the MCHC decrease is a compensatory mechanism to ensure the preservation of the erythrocytes (Figure 4), in view of the elevated pressure present in the disease, ensuring the preservation of the pregnant woman's life.

Returning to the mechanical selection hypothesis, it is possible that the criterion for selection is exactly the hemoglobin concentration. The erythrocytes with higher hemoglobin content would offer more resistance and would be more vulnerable to damage and removal, leaving unchanged the erythrocyte populations with lower hemoglobin content and with greater deformability and stability both in the presence of mechanical aggression *in vivo* and in the presence of aggression by osmotic expansion in a low osmolarity medium *in vitro* (Figure 4).

The absence of a significant correlation between MCHC and indirect bilirubin (*ρ* = −0.033; *p*=0.932) and total bilirubin (*ρ* = −0.336; *p*=0.262) in EOPE should mean the existence of a much more complex environment in this condition, with a larger group of factors governing bilirubin levels. This is in fact true in light of the interference of oxidative stress on the deformability, stability, and lysis of erythrocytes, on the one hand, and the interference of hepatic dysfunction on bilirubin levels, on the other hand. Indeed, a significant and strong positive correlation was observed between AST and t-B levels (*ρ* = +0.500; *p*=0.019) in EOPE.

Although erythropoiesis accelerates in normal gestation, with increased reticulocyte release in the circulation [52], erythropoiesis becomes even more accelerated in EOPE, as can be inferred from the higher values observed in the reticulocyte index of EOPE group with respect to C group. After a few days in the bloodstream, reticulocytes completely lose their organelles and acquire the biconcave disc format that is typical of young mature erythrocytes. For this reason, the erythrocyte population in gestation is predominantly composed of young cells [47], which would be osmotically more fragile [48], due to the occurrence of progressive increase in the sphericity of the erythrocyte, with reduction of diameter and thickening of the membrane during pregnancy [47, 53]. Indeed, the positive correlation observed between *H*_50_ and reticulocyte index (*ρ* = +0.524; *p*=0.009) in the control group means that *in vitro* the osmotic stability of erythrocytes decreased with increasing reticulocyte ratio in the blood of healthy pregnant women in this study. But there are reports that reticulocytes show a 10% increase in their membrane stability when compared to young erythrocytes [54]. However, due to the noninclusion of a group of nonpregnant women, the present study could not determine the influence of gestation on the membrane stability of erythrocytes. Given the complex and nonlinear nature of the relationships between the variables of the broad set of factors governing osmotic stability of erythrocytes, it is difficult to establish inferences exclusively from bivariate correlations.

Even so, some significant correlations deserve to be highlighted, especially when associated with situations where there has been greater variation, as is the case of the EOPE group in relation to normal pregnancy. In the EOPE group, in which there was an acceleration of erythropoiesis associated with hepatic dysfunction, the correlations presented by RDW are noteworthy. RDW showed significant negative correlations with TGC (*ρ* = −0.661; *p*=0.019), t-C (*ρ* = −0.749; *p*=0.005), LDL-C (*ρ* = −0.612; *p*=0.046), and urea (*ρ* = −0.601; *p*=0.030), and a positive correlation with 1/*H*_50_ (*ρ* = +0.658; *p*=0.028). Although high levels of RDW have been associated with excess membrane cholesterol content [55] and excessively high blood levels of cholesterol have been associated with the generation of hemolytic anemia and morphological atypia [31, 56], the relationship of RDW with cholesterolemia should not be linear [31]. Particularly in view of the increase in reticulocyte population, another aspect of the influence of blood cholesterol levels should be manifested. This is why in the EOPE group, RDW was negatively associated with TGC, t-C, and LDL-C, as previously affirmed. These correlations make even more sense in light of the possible meaning of the also negative correlation observed between RDW and urea (*ρ* = −0.601; *p*=0.030). Although high levels of urea in the EOPE group are associated with a decline in renal function, lower levels of urea also signal the occurrence of hepatic dysfunction, which means less production of lipids. Thus, higher urea levels would also mean higher hepatic triglyceride and cholesterol production and lower RDW values due to the hematologic context present in this group, that is, in the presence of an increased reticulocyte population. On the other hand, the positive correlation observed between RDW and 1/*H*_50_ (*ρ* = +0.658; *p*=0.028) shows that for the range of values of this variable presented by the EOPE group, the increase in RDW was associated with an increase in erythrocyte stability. In relation to the variability existing between these parameters, it is difficult to infer what is desirable and what is undesirable because each of these variables or properties is undergoing the simultaneous influence of other variables.

This is the case of erythrocyte membrane stability, which in LOPE showed to be inversely associated with MCHC (due to the positive correlations presented by *H*_50_ (*ρ* = +0.465; *p*=0.039) and *H*_100_ (*ρ* = +0.647; *p*=0.002) with MCHC) and MCV values (due to the negative correlations presented by variables *dX* (*ρ* = −608; *p*=0.27), *dX*/*A*_min_ (*ρ* = −0.731; *p* < 0.001), and *dX*/*H*_50_ (*ρ* = −0.666; *p*=0.001) with MCV). Since MCV is governed by MCH, which is quite obvious and can be confirmed by the strong correlations observed between MCV and HCM in the three groups of this study, it seems that the increase in erythrocyte stability with the decrease in volume is due to a decrease in hemoglobin content. This makes a lot of sense, both *in vivo*, in which the decrease in hemoglobin content decreases cytosolic viscosity and raises the deformability and mechanical stability of erythrocytes [54], and *in vitro*, a situation in which the reduction in hemoglobin content decreases the osmotic pressure associated with the entry of water into the cell, especially in view of the hyposmotic condition in the external aqueous medium.

In the LOPE group, significantly higher values of weight gain and BMI in relation to the other groups suggest the participation of obesity in the etiology of the disease. In fact, higher adiposity is associated with chronic inflammation, ROS release, and membrane lipoperoxidation, thus generating structural and functional damages [57, 58]. Thus, the increase in lipid peroxidation, due to obesity, could trigger the disease, even in the presence of adequate placentation. In the LOPE group, the association between BMI and the osmotic stability index *dX*/*H*_50_ (*ρ* = +0.571; *p*=0.041) suggests the influence of obesity, probably due to lipoperoxidation and dyslipidemia, in increasing erythrocyte membrane stability. This type of effect exerted by blood lipids should not be surprising since transfer of cholesterol to the erythrocyte membrane has dual effects on the stability and functionality of these cells [31].

In the presence of a hypoxemic environment, there is a reduction in peripheral vascular resistance and a compensatory increase in local flow velocity. It seems that a similar mechanism occurs in the cerebrovascular microcirculation of volunteers with EOPE, as may be inferred from the behavior of some hemodynamic indices of the OA.

Positive correlations of RI with erythrocyte counts (*ρ* = +0.694; *p*=0.026) and hemoglobin levels (*ρ* = +0.702; *p*=0.024) show that the lower RI values observed in this group are associated with lower red-cell counts, which would require a compensatory increase in local flow velocity. Indeed, the PR increase that was observed in the EOPE group and the negative correlation observed between PR and hemoglobin (*ρ* = −0.669; *p*=0.035), also in this group, indicate that the blood flow changes observed in OA are associated with changes in hemoglobin levels.

On the other hand, exacerbation of cerebral vasodilation is responsible for loss of self-regulation, extravasation of plasma, and development of posterior reversible encephalopathy, known as PRES [14] and considered the terminal stage of preeclampsia.

The negative association observed between placental weight and MCV (*ρ* = −0.618; *p*=0.004), MCH (*ρ* = −0.458; *p*=0.042) and MCHC (*ρ* = −0.519; *p*=0.019) indices in the LOPE group indicates that the mechanical selection of smaller erythrocytes, with lower hemoglobin content, is associated with better gestational outcome. A similar protective phenomenon appears to occur in the central territory since smaller red cells and lower hemoglobin levels were associated with lower central flow and thus lower the risk of hydrostatic edema of the cerebral parenchyma, as can be inferred from the negative correlation observed between IR and MCV (*ρ* = −0.642; *p*=0.045).

Renal injury in preeclampsia is marked by increased urea and, mainly, creatinine. The elevation of these substances is related to an increase in blood pressure and a hyperdynamic cardiovascular state. Thus, there will be increased pulse pressure and, consequently, increased flow velocity of the ophthalmic artery. Indeed, this is what can be inferred by the positive associations of P2 with creatinine (*ρ* = +0.667; *p*=0.050) and urea (*ρ* = +0.697; *p*=0.025) and of PR with creatinine (*ρ* = +0.753; *p*=0.019) in the EOPE group.

In EOPE, the positive correlation observed between PR and VLDL-C (*ρ* = −0.904; *p*=0.001), lipoprotein that was associated with endothelial dysfunction [59], indicates its participation in peripheral resistance and elevation of blood pressure levels, with a consequent increase in pulse pressure and in the systolic peaks of the ophthalmic artery.

In the present study, PR values, the main indicator of changes in flow velocity in the Doppler velocimetry of the OA (Figure 3), differentiated the cases of PE in relation to the normotensive patients but did not demonstrate a significant difference between the LOPE and EOPE groups. In practice, PR has the power to detect PE when above 0.78 [15]. The significant difference found between P2 of the EOPE group, but not of the LOPE group, in relation to the C group (Table 1), suggests that P2 could also be used to differentiate the vascular behavior of EOPE in relation to LOPE. In fact, P2 values above 24.6 cm/s in the first trimester of gestation were associated with a prediction of the risk of developing EOPE [60].

It is possible that the stratification of PE on the basis of a vascular referential capable of differentiating EOPE from LOPE could better clarify the differences in the complex relationships between the anthropometric, hemodynamic, hematologic, and biochemical profiles of these two forms of preeclampsia.

It is important to emphasize that the control group used here may be a limitation of this study since this group consisted of volunteers who had full-term gestation, which may raise doubts as to whether the hematologic findings found in the EOPE group can in fact be attributed to preeclampsia or whether they are inherent to existing differences in gestational age between the two groups, where there is a single reference range for each hematologic and biochemical variable for the whole third trimester of gestation [61].

## Figures and Tables

**Table 1:** Comparisons of the values of variables from normotensive pregnant women and pregnant women with late-onset preeclampsia (LOPE) and early-onset preeclampsia (EOPE) at the third trimester of pregnancy.

Variables	Normotensive	LOPE	EOPE
Maternal age (y)^†^	25.0 ± 10.5 (28)	26.0 ± 14.0 (23)	27.0 ± 10.0 (13)
Weight gain (kg)^§^	10.0 ± 3.0 (26)^a^	20.0 ± 7.0 (9)^a,b^	14.0 ± 5.0 (5)^b^
BMI (pregestational) (kg/m^2^)^§^	24.2 ± 5.3 (27)	25.16 ± 10.3 (10)	23.3 ± 5.5 (7)
BMI (kg/m^2^)^§^	29.2 ± 5.1 (27)^a^	32.0 ± 8.4 (13)^a^	29.7 ± 6.2 (6)
Gestational age (w)^§^	40.0 ± 1.0 (29)^a,b^	38.0 ± 2.0 (20)^b,c^	31.5 ± 4.0 (13)^a,c^
Birth weight (kg)^§^	3.5 ± 0.5 (28)^a,b^	2.9 ± 0.6 (22)^b,c^	1.8 ± 0.9 (11)^a,c^
Growth centile (%)			
<10^∗^	0% (0)	4.3% (1)	23.1% (3)
10–50^∗^	21.0% (6)	39.1% (9)	38.5% (5)
50–90^∗^	65.5% (19)	39.1% (9)	7.7% (1)
>90^∗^	10.3% (3)	4.3% (1)	15.4% (2)
Unknown	3.4% (1)	13.0% (3)	15.4% (2)
Placenta weight (g)^§^	590.0 ± 80.0 (28)^a^	512.5 ± 160.0 (22)^b^	337.5 ± 200.0 (12)^a,b^
SBP (mmHg)^§^	120.0 ± 10.0 (28)^a,b^	150.0 ± 30.0 (23)^a^	150.0 ± 20.0 (13)^b^
DBP (mmHg)^§^	70.0 ± 10.0 (28)^a,b^	100 ± 20.0 (23)^a^	100.0 ± 10.0 (13)^b^
RBC (10^6^ cells/mm^3^)^†^	4.15 ± 0.57 (29)^a^	4.08 ± 0.44 (22)	3.78 ± 0.68 (13)^a^
Ht (%)^†^	34.6 ± 3.8 (29)	34.7 ± 5.6 (22)	33.2 ± 3.8 (13)
Hb (g/dL)^†^	12.0 ± 1.5 (29)	11.8 ± 1.8 (22)	11.3 ± 1.3 (13)
MCV (fL)^§^	85.3 ± 6.4 (29)	82.9 ± 8.9 (21)	87.3 ± 6.1 (13)
MCH (pg)^§^	29.5 ± 2.7 (29)	27.9 ± 3.7 (21)	29.8 ± 2.7 (13)
MCHC (g/dL)^§^	34.1 ± 1.6 (29)	33.8 ± 1.5 (21)	33.9 ± 2.3 (13)
RDW (%)^§^	13.3 ± 1.3 (29)	14.0 ± 1.9 (21)	13.0 ± 1.3 (13)
Rtc index (%)^§^	1.1 ± 1.4 (27)^a^	1.4 ± 1.1 (21)	1.7 ± 1.6 (11)^a^
Plt (10^3^ cells/mm^3^)^§^	229.0 ± 7.0 (29)^a^	213.5 ± 102 (22)^b^	141.0 ± 52.0 (13)^a,b^
MPV (fL)^§^	11.0 ± 2.0 (29)	10.2 ± 2.4 (21)	10.0 ± 2.3 (13)
t-C (mg/dL)^§^	219.2 ± 65.0 (27)	198.0 ± 59.0 (19)	227.0 ± 61.9 (12)
HDL-C (mg/dL)^§^	59.8 ± 28 (27)	59.9 ± 13.3 (21)	60.3 ± 20.5 (12)
VLDL-C (mg/dL)^§^	40.0 ± 16.0 (28)	40.5 ± 19.8 (16)	47.0 ± 31.2 (11)
LDL-C (mg/dL)^§^	131.5 ± 63.3 (28)	106.0 ± 54.0 (17)	113.5 ± 66.3 (11)
TGC (mg/dL)^§^	203.0 ± 111.0 (28)	210.0 ± 128.0 (17)	238.0 ± 121.8 (12)
Proteinuria (0^+^)^∗^	100.0% (29)^a,b^	31.3% (5)^a^	0% (0)^b^
Proteinuria (1^+^)^∗^	0% (0)	18.8% (3)	22.2% (2)
Proteinuria (2^+^)^∗^	0% (0)	37.5% (6)	44.4% (4)
Proteinuria (3^+^)^∗^	0% (0)	12.5% (2)	11.1% (1)
Proteinuria (4^+^)^∗^	0% (0)	0% (0)	22.2% (2)
Proteinuria (unknown)	0% (0)	30.4% (7)	30.7% (4)
ALT (U/L)^§^	14.9 ± 2.9 (29)^a,b^	17.0 ± 11.0 (23)^a^	27.0 ± 27.7 (13)^b^
AST (U/L)^§^	9.0 ± 3.2 (28)^a,b^	12.0 ± 7.0 (23)^a^	22.0 ± 45.8 (13)^b^
LDH (U/L)^§^	193.0 ± 39.0 (27)^a,b^	236.0 ± 49.0 (23)^a,c^	292.0 ± 274.0 (13)^b,c^
HSA (g/dL)^§^	3.5 ± 0.4 (25)^a^	3.3 ± 0.6 (15)^b^	3.0 ± 0.6 (11)^a,b^
Urea (mg/dL)^†^	14.6 ± 3.6 (29)^a,b^	21.0 ± 10.9 (23)^a,c^	35.0 ± 19.0 (13)^b,c^
Cn (mg/dL)^§^	0.58 ± 0.21 (29)^a^	0.70 ± 0.2 (23)	0.78 ± 0.29 (12)^a^
Uric acid (mg/dL)^§^	4.3 ± 1.0 (29)^a,b^	5.4 ± 1.4 (23)^a,c^	7.6 ± 1.4 (13)^b,c^
i-B (mg/dL)^§^	0.205 ± 0.13 (26)	0.17 ± 0.23 (16)	0.220 ± 0.480 (11)
t-B (mg/dL)^§^	0.425 ± 0.19 (26)	0.28 ± 0.17 (20)	0.340 ± 0.490 (13)
Na (mEq/L)^§^	138.0 ± 2.5 (24)^a^	140.0 ± 4.0 (17)^c^	136.0 ± 5.0 (13)^a,c^
K (mEq/L)^§^	4.05 ± 0.42 (25)^a^	4.0 ± 0.5 (17)	4.56 ± 0.47 (13)^a^
Fe (mg/dL)^†^	78.0 ± 46.0 (27)^a^	84.9 ± 46.1 (20)^b^	106.5 ± 89.3 (12)^a,b^
Ferritin (ng/mL)^§^	26.5 ± 14.64 (25)^a^	31.5 ± 32.5 (17)^b^	156.2 ± 244.7 (12)^a,b^
RI^§^	0.78 ± 0.04 (26)^a,b^	0.73 ± 0.09 (17)^a^	0.63 ± 0.08 (10)^b^
PI^§^	1.96 ± 0.44 (26)^a,b^	1.62 ± 0.46 (17)^a^	1.14 ± 0.87 (10)^b^
PSV (cm/seg)^§^	31.4 ± 7.4 (27)	30.6 ± 9.8 (17)	30.0 ± 15.0 (10)
P2 (cm/seg)^§^	17.9 ± 10.7 (27)^a^	22.3 ± 4.1 (17)	25.3 ± 14.2 (10)^a^
PR^§^	0.58 ± 0.13 (27)^a,b^	0.71 ± 0.27 (17)^a^	0.85 ± 0.19 (10)^b^
*V*_mean_ (cm/seg)^†^	12.5 ± 4.9 (26)	13.3 ± 4.8 (15)	16.2 ± 7.1 (7)
EDV (cm/seg)^§^	7.1 ± 3.7 (27)	8.2 ± 3.2 (17)	10.8 ± 5.4 (9)
*A*_min_ (∆OD)^§^	1.060 ± 0.148 (26)	1.032 ± 0.237 (22)	0.099 ± 0.074 (11)
*A*_max_ (∆OD)^§^	0.019 ± 0.021 (26)	0.019 ± 0.032 (22)	0.009 ± 0.009 (11)
*H*_0_ (g/dL NaCl)^†^	0.480 ± 0.031 (26)^d^	0.472 ± 0.048 (22)	0.458 ± 0.028 (11)^d^
*H*_50_ (g/dL NaCl)^§^	0.447 ± 0.036 (26)^a^	0.437 ± 0.037 (22)^d^	0.417 ± 0.028 (11)^a,d^
*H*_100_ (g/dL NaCl)^§^	0.423 ± 0.030 (26)^a^	0.402 ± 0.034 (22)^d^	0.388 ± 0.013 (11)^a,d^
1/*H*_50_ (g/dL NaCl)^−1§^	2.236 ± 0.177 (26)^a^	2.283 ± 0.201 (22)^d^	2.400 ± 0.161 (11)^a,d^
*H*_50_ (g/dL NaCl)^§^	0.447 ± 0.036 (26)^a^	0.437 ± 0.037 (22)^d^	0.417 ± 0.028 (11)^a,d^
*dX* (g/dL NaCl)^§^	0.017 ± 0.027 (26)	0.015 ± 0.007 (22)	0.016 ± 0.010 (11)
*dX*/*H*_50_^§^	0.038 ± 0.208 (26)	0.035 ± 0.018 (22)	0.038 ± 0.022 (11)
*dX*/*A*_min_^§^	0.016 ± 0.007 (26)	0.014 ± 0.011 (22)	0.018 ± 0.011 (11)

The values are presented as ^∗^frequency, ^§^median ± IQR (N), or †mean ± SD (N). ^a,b,c,d^Statistically significant (*p* < 0.05) and borderline differences (0.05 < *p* < 0.10), respectively, when present as pairs of common letters. Comparisons were done by chi-square test for ^∗^categorical data, Kruskal–Wallis with Dunn–Bonferroni post hoc test for ^§^nonnormally distributed data, or ANOVA with Bonferroni post hoc test for †normally distributed data. SBP, systolic blood pressure; DBP, diastolic blood pressure; Ht, hematocrit; Hb, hemoglobin; RBC, erythrocytes; MCV, mean corpuscular volume; MCH, mean corpuscular hemoglobin; MCHC, mean corpuscular hemoglobin concentration; RDW, red-cell distribution width; Rtc index, reticulocyte index; Plt, platelets; MPV, mean platelet volume; t-C, total cholesterol; HDL-C, high-density lipoprotein cholesterol; VLDL-C, very-low-density lipoprotein cholesterol; LDL-C, low-density lipoprotein cholesterol; TGC, triglycerides; AST, aspartate aminotransferase; ALT, alanine aminotransferase; LDH, lactate dehydrogenase; HSA, human serum albumin; Cn, creatinine; i-B, indirect bilirubin; t-B, total bilirubin; Na^+^, sodium; K^+^, potassium; Fe, serum iron; RI, resistance index; PI, pulsatility index; PSV, peak systolic velocity; P2, second peak of systolic velocity; PR, peak ratio; *V*_mean_, mean velocity; EDV, end-diastolic velocity; *A*_min_, absorbance at 540 nm associated with residual lysis of the erythrocytes population; *A*_max_, absorbance at 540 nm associated with lysis of the whole population of erythrocytes; *H*_0_, saline concentration where *in vitro* hemolysis begins; *H*_50_, saline concentration capable of promoting 50% hemolysis, *H*_100_, saline concentration where *in vitro* lysis ends; 1/*H*_50_, inverse NaCl concentration capable of promoting 50% hemolysis; *dX*, variation in the concentration of NaCl responsible for total hemolysis.

## Additional Points

*Highlights*. (i) Late-onset preeclampsia was associated with obesity. (ii) Early-onset preeclampsia was associated with increased osmotic stability of erythrocyte membrane. (iii) Greater maternal and fetal protection in preeclampsia was associated with mechanical selection of smaller erythrocytes and with lower hemoglobin content. (iv) Doppler velocimetry of ophthalmic artery in early-onset preeclampsia but not late-onset preeclampsia was associated with increase in the second systolic peak.
